# Amino Acid Metabolism of the Skin: Control by Specific Enzymes and Contribution to Protective Functions

**DOI:** 10.3390/metabo15090601

**Published:** 2025-09-09

**Authors:** Corina Dörner, Julia Steinbinder, Attila Placido Sachslehner, Supawadee Sukseree, Leopold Eckhart

**Affiliations:** Department of Dermatology, Medical University of Vienna, 1090 Vienna, Austria

**Keywords:** skin, epidermis, arginase, histidase, urocanic acid, tyrosinase, melanin, keratinocytes, melanocytes, amino acid transporter

## Abstract

The skin protects the body from damaging external stressors. The function of its outermost compartment, the epidermis, depends on high rates of protein synthesis and the production of protective molecules, both requiring amino acids as precursors. Conversely, the degradation of the epidermal barrier protein filaggrin releases free amino acids. Here, we review the epidermal amino acid metabolism, focusing on the metabolism of histidine, arginine and tyrosine, which are subjected to epidermal cell-specific control mechanisms. Histidine and arginine are metabolized by enzymes that are transcriptionally upregulated during terminal differentiation of keratinocytes, while tyrosine is specifically metabolized in melanocytes. Arginase converts arginine into ornithine and urea. While ornithine is decarboxylated to putrescine, a regulator of cellular proliferation, urea contributes to the moisturization of the skin surface. Histidase, also known as histidine ammonia lyase, converts histidine into urocanic acid (UCA) and ammonia. UCA is the main ultraviolet-absorbing molecule of the cornified layer of the epidermis, serving as a natural sunscreen of human skin. In melanocytes, tyrosinase initiates the polymerization of tyrosine to melanin, the main skin pigment that absorbs both visible light and ultraviolet radiation. The current evidence indicates that the metabolism of histidine, arginine, tyrosine and other amino acids critically influences normal and diseased skin.

## 1. Introduction

Amino acids are organic molecules that contain both an amino group (−NH_2_) and a carboxyl group (−COOH). They play central roles in living organisms and also in all organs of the human body, including the skin. The aim of this article is to review the turnover of amino acids in the skin with a focus on its outermost compartment, the epidermis, which directly interacts with the environment. We provide a general introduction to amino acids before we describe and discuss the peculiarities of the amino acid metabolism in epidermal keratinocytes and melanocytes.

Twenty alpha-amino acids, in which the amino group and the carboxyl group are attached to the same carbon atom, are incorporated into proteins according to the genetic code of mRNAs. These standard proteinogenic amino acids are alanine, cysteine, aspartic acid, glutamic acid, phenylalanine, glycine, histidine, isoleucine, lysine, leucine, methionine, asparagine, proline, glutamine, arginine, serine, threonine, valine, tryptophan and tyrosine. The essential amino acids histidine, isoleucine, leucine, lysine, methionine, phenylalanine, threonine, tryptophan and valine must be taken up from the diet, whereas the others can be synthesized in sufficient quantities. The non-proteinogenic amino acids of the human body are functionally diverse and include intermediates of biosynthetic pathways such as delta-aminolevulinic acid, products of post-translational modifications of proteins such as citrulline, neurotransmitters such as gamma-aminobutyric acid (GABA), regulators of bioenergetics such as creatine, and others [1].

The metabolism of amino acids is partly controlled by specific enzymes that either synthesize or degrade amino acids and transporters that determine their localization within the tissue and in subcellular compartments [2,3,4,5]. Importantly, the pool of amino acids is affected by complex cellular processes such as ribosomal protein synthesis (translation), proteasomal degradation of proteins and lysosomal protein degradation [6]. At the organismal level, the uptake of amino acids as nutrients and the control of amino acid levels in the circulation are tightly controlled and important for the homeostasis of amino acids at the levels of organs, tissues and cells.

The basic structure and functions of the skin are well defined and have been reviewed extensively. An epithelium, the epidermis, forms a major barrier of the body against the environment [7,8,9,10]. Underneath, the dermis and hypodermis supply the epidermis with nutrients and contribute to the mechanical stability and wound healing potential of the skin [11,12]. The complexity of the skin arises from the presence of multiple cell types and the dynamic interactions with other organs and the environment [13,14,15].

The turnover of amino acids is important in all compartments of the skin [16,17], and research has defined different roles of amino acids in the various cell types. For example, experimental studies suggest that the production of collagen by dermal fibroblasts is partly controlled by the availability of proline [18,19]. Glutamic acid released from neurons suppresses mast cell hyperresponsiveness and skin inflammation to maintain the immune homeostasis of the skin [20]. Exogenous cystine, the oxidized derivative of the amino acid cysteine, reduces lipid peroxidation and suppresses ferroptotic cell death in Th9 cells [21]. Dietary serine levels affect the response to injury of hair follicle stem cells, with deprivation of serine leading to re-epithelialization and skin repair instead of hair growth [22]. Interestingly, the absence of hair follicles and increased epidermal thickness on the skin of palms and soles are associated with unique features of the amino acid turnover, as suggested by gas chromatography-time-of-flight/mass spectrometry-based metabolomics [23]. Furthermore, it is important to note that microbes contribute to the metabolism of amino acids in healthy and diseased skin [24,25,26,27].

**Figure 1:**
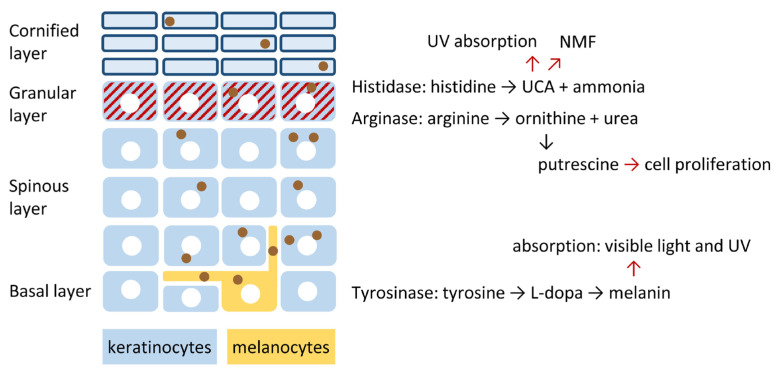
The epidermal amino acid metabolism features three reaction pathways that are not active in most other tissues. A simplified diagram schematically depicts the cellular architecture of the epidermis. Keratinocytes undergo differentiation as they move upward while forming distinct layers. White circles indicate nulcei. Melanocytes produce melanin-containing vesicles, the melanosomes (depicted as brown circles), which are transferred to keratinocytes. The enzymes histidase and arginase are active in the granular layer (red-striated) while tyrosinase is active in melanocytes. The reactions catalyzed by these enzymes lead to the conversion of histidine, arginine and tyrosine into bioactive molecules that contribute to the function of the epidermis as a protective and self-regenerating tissue.

Here, we highlight parts of the cutaneous amino acid metabolism that are specific to one or a few cell types or cell differentiation states. The amino acid metabolism of the skin is compartmentalized and involves both pathways active in all or most cells and others that are unique to one cell type or one differentiation stage. This review article focuses on the enzymatic turnover of arginine, histidine and tyrosine in the epidermis (Figure 1). The metabolism and transport of other amino acids are addressed in Section 6 and Section 7 of the article.

## 2. Specific Epidermal Expression Patterns of Arginase, Histidase and Tyrosinase Are Hallmarks of the Normal Human Skin

**Figure 2:**
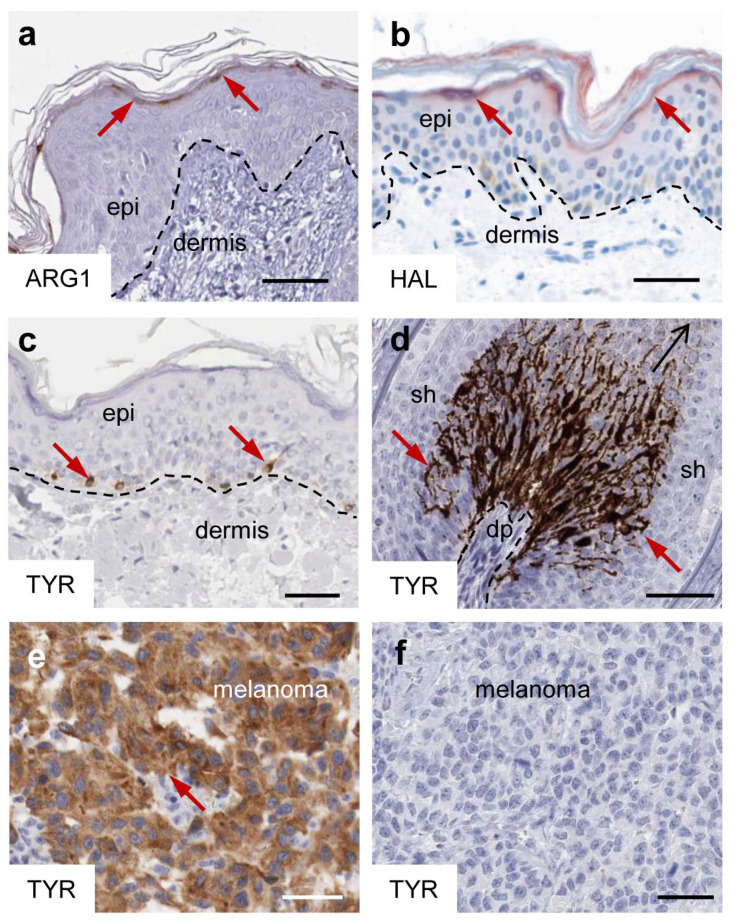
Expression patterns of arginase, histidase and tyrosinase. Immunostaining of arginase 1 (ARG1) (**a**), histidase/histidine ammonia lyase (HAL) (**b**), tyrosinase (TYR) (**c**–**f**) in the skin (**a**–**c**), hair follicle (**d**) and melanomas (**e**,**f**). Expression sites are indicated by red arrows. The broken lines in panels a–d mark the dermo-epidermal junction. dp, dermal papilla; epi, epidermis; sh, sheath. The images are reproduced with permission from (**b**) [28] and (**a**,**c**–**f**) the open access database Protein Atlas (https://www.proteinatlas.org/ENSG00000118520-ARG1/tissue/skin#img, last accessed on 23 June 2025; https://www.proteinatlas.org/ENSG00000077498-TYR/tissue/skin#img, last accessed on 23 June 2025; https://www.proteinatlas.org/ENSG00000077498-TYR/cancer/melanoma#img, last accessed on 23 June 2025) [29]. Scale bars, 50 µm.

The enzymes histidase, arginase and tyrosinase, catalyzing reactions of histidine, arginine and tyrosine, respectively, have in common a profoundly non-homogeneous distribution in the epidermis with high abundance in particular cells. Immunohistochemical analyses demonstrate that arginase 1 and histidase, also known as histidine ammonia lyase (HAL), are expressed in keratinocytes of the epidermal granular layer (Figure 2a,b), whereas tyrosinase is expressed in melanocytes residing in the basal layer of the epidermis and in the keratogenous zone of hair follicles (Figure 2c,d).

The expression of these enzymes is altered in diseased skin and tumors arising from epidermal cells. Arginase 1 is upregulated in psoriasis [30,31,32]. Missense mutations of the *HAL* gene cause histidinemia, which is the most frequent inborn metabolic error in Japan [33]. Homozygous or compound heterozygous mutations of *Tyrosinase* (*TYR*) cause oculocutaneous albinism type 1A [34,35]. The expression of tyrosinase is either maintained or lost after malignant transformation of melanocytes, leading to tyrosinase-positive and tyrosinase-negative melanoma metastasis (Figure 2e,f) [36]. Importantly, the activator of tyrosinase expression, the microphthalmia-associated transcription factor (MITF), plays oncogenic roles in melanoma [37].

## 3. Arginase Converts Arginine into Ornithine: Mechanism and Function

### 3.1. Arginase: Expression, Regulation and Enzymatic Activity in the Skin

Arginase is a manganese-dependent metalloenzyme that catalyzes the hydrolytic conversion of arginine into ornithine and urea. Mammals have two isoforms, arginase 1 and 2, which are encoded by distinct genes, *ARG1* and *ARG2* [38]. *ARG1* is predominantly expressed in the liver and at lower levels in the bone marrow and in the skin. In the liver, *ARG1* controls a critical step of the urea cycle, which converts toxic ammonia to urea [38]. *ARG2* has a broader expression pattern in the organs, with peak levels in the thyroid and prostate. It is mainly expressed in smooth muscle cells [38]. ARG1 is the predominant isoform of arginase in normal skin where it is expressed constitutively in the granular layer of the epidermis (Figure 2a). ARG1 is further upregulated in psoriatic skin with expression being detectable in all epidermal layers [30]. The expression of *ARG2* is induced during skin wound healing [39], although ARG1 is more critical for skin repair [40,41].

**Figure 3:**
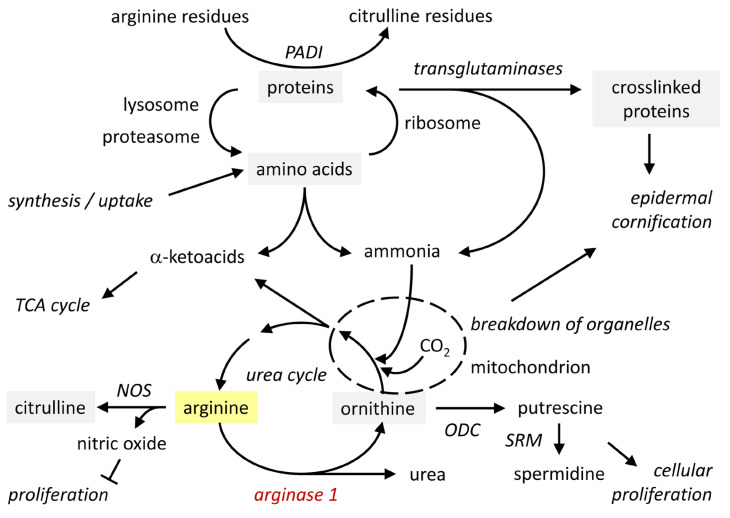
The role of arginase in the urea cycle and links to processes active in epidermal keratinocytes. Schematic and simplified overview of the arginine metabolism in epidermal keratinocytes. ODC, ornithine decarboxylase; NOS, nitric oxide synthase; PADI, peptidyl arginine deiminase; SRM, spermidine synthase; TCA, tricarboxylic acid cycle.

Arginase activity can be isolated from the cornified layer (stratum corneum) of the epidermis [42,43]. Immunohistochemistry detects ARG1 only in the granular layer, suggesting that cornification leads to presumably transglutaminase-mediated epitope masking as it is known for other antigens [44]. ARG1 is catalytically active at the physiological pH 7.4, but its optimum is found at pH 9.5 [45]. As the pH and the concentration of manganese ions are suboptimal in the stratum corneum [42,46], the detection of catalytic activity under assay conditions does not necessarily correlate with activity in situ. Interestingly, the concentration of arginine increases from the lower to the middle layer of the stratum corneum and this increase depends on the presence of filaggrin, suggesting that proteolytic degradation of filaggrin releases arginine [47]. In the uppermost layer of the stratum corneum, the concentration of arginine decreases again. The pH is acidic in mid-stratum corneum (pH 5.4) and increases to close-to-neutral (pH 6.7) towards the surface [46,48]. Thus, it is conceivable that in the outermost layers of the stratum corneum ARG1 converts arginine to ornithine and urea, which act as components of the natural moisturizing factor (NMF) [49,50]. The metabolic pathways depending on arginine are summarized in Figure 3.

### 3.2. Functions of Arginine and Its Metabolites, Ornithine and Urea

Arginine is a proteogenic amino acid in all skin cells. In differentiated keratinocytes of the epidermis, many arginine residues of structural proteins are deiminated to citrulline residues under the control of peptidyl arginine deiminases (PADIs) 1 and 3 [51]. Besides its role in protein synthesis, arginine is the substrate of arginases and nitric oxide synthases (NOS). Arginase activity generates ornithine, which can be converted to putrescine under catalysis by ornithine decarboxylase (ODC) and subsequently transformed into spermidine and spermine. The latter are polyamines that affect cell proliferation and differentiation in the epidermis and other tissues [40,52,53]. Nitric oxide synthases (NOS) convert arginine into citrulline and NO in a reaction that depends on molecular oxygen (O_2_) for oxygenation and nicotinamide adenine dinucleotide phosphate (NADP)-H as reducing agent. All three NOS isoforms were reported to be expressed in epidermal keratinocytes [54], with inducible NOS (iNOS), encoded by the gene *NOS2*, likely playing the main role [52]. NO is a signaling molecule that increases vasodilation and contributes to the killing of pathogens. Thus, arginase and NOS compete for the same substrate, arginine. The alternative routes of utilizing arginine are important in macrophages. M1 macrophages express high levels of inducible nitric oxide synthase (iNOS) to achieve their function in the defense against pathogens. By contrast, M2 macrophages express high levels of ARG1 to promote cell proliferation and tissue repair [55].

Dysregulation of arginase 1 in skin diseases is suggested to cause defects via direct effects on the levels of ornithine and urea as components of the NMF and indirect effects on the levels of ornithine-derived polyamines and NO. The production of polyamines is enhanced by arginase activity, whereas NO production is decreased due to substrate competition with NOS. In psoriasis, the expression of ARG1 is increased and changed in its pattern within the epidermis [30,31]. Instead of prominent expression exclusively in the granular layer, ARG1 is widely expressed in the increased number of epidermal cell layers [30]. The conversion of arginine to ornithine and polyamines such as spermidine enhanced skin inflammation in a mouse model of psoriasis, whereas arginase inhibitors nor-NOHA and DFMO reduced the severity of the disease symptoms in these mice [32]. In atopic dermatitis, the downregulation of ARG1 has been suggested to impair terminal differentiation of keratinocytes and to increase the susceptibility to infection [52]. Ornithine is elevated in the blood of patients having rosacea, which has been linked to an aberrant amino acid metabolism [56]. However, arginase activities remain to be investigated in this disease.

## 4. Histidase Converts Histidine into Urocanic Acid: Mechanism and Function

### 4.1. Histidase: Expression, Regulation and Enzymatic Activity in the Skin

Histidine is converted into urocanic acid in a reaction that is catalyzed by histidine ammonia lyase (HAL), which is commonly known as histidase [57,58]. The *HAL* gene is located on human chromosome 12q23.1. Its main expression sites are the liver and the epidermis [59,60]. In the liver, the transcription of *HAL* is regulated by the availability of histidine derived from the diet [61]. In the epidermis, the expression of *HAL* is induced by differentiation of keratinocytes [62]. Histidase locates to the cytosol where it catalyzes the non-oxidative deamination of L-histidine.

The epidermal expression of histidase is confined to the granular layer of the epidermis (Figure 2b). In cultured keratinocytes, the transcription of the *HAL* gene is induced by differentiation of keratinocytes upon prolonged maintenance in confluent culture and culture in skin equivalent models [62]. Transcription factor binding sites have been identified in the promoter of *HAL* [60], but the mechanisms of regulation are not known at present.

The protein histidase is detected immunohistochemically in the granular and cornified layers of the epidermis [28] and by mass spectrometry-based proteomics of the stratum corneum [63]. The catalytic activity of histidase is detectable in the superficial layers [64]. However, due to the decrease in the pH upon cornification [46], histidase is considered inactive in most of the cornified layer [64].

The activity of histidase is modified in skin diseases. In psoriasis, histidase activity was reported to be elevated along with increased concentrations of its product, UCA [65,66]. The pro-inflammatory cytokines interleukin 1 (IL-1) and tumor necrosis factor (TNF)-α reduce histidase expression levels in keratinocytes [62]. Likewise, treatment with retinoic acid [62] and 8-methoxypsoralen [67] suppress the expression of *HAL* [62].

Loss-of-function mutations of *HAL* cause histidinemia (Online Mendelian Inheritance in Man, OMIM #235800, https://omim.org/entry/235800, last accessed on 27 June 2025) [68]. This disorder has a high prevalence (1:8,400) in Japan [60]. Loss-of-function alleles of *HAL* are associated with a lower risk of coronary heart disease, possibly due to elevated concentrations of histidine in the serum [69]. Histidinemic individuals have decreased levels of UCA in the blood and the stratum corneum [70]. A mouse model of histidinemia, the so-called Peruvian mice [71,72], is defined by a point mutation (R322Q) of the histidase gene, resulting in destabilization of the protein. In human carriers of *HAL* mutations, higher levels of histidine in the blood appear to undergo decarboxylation to yield histamine, which subsequently increases the risk of skin inflammation [73,74].

### 4.2. Histidine and UCA

**Figure 4:**
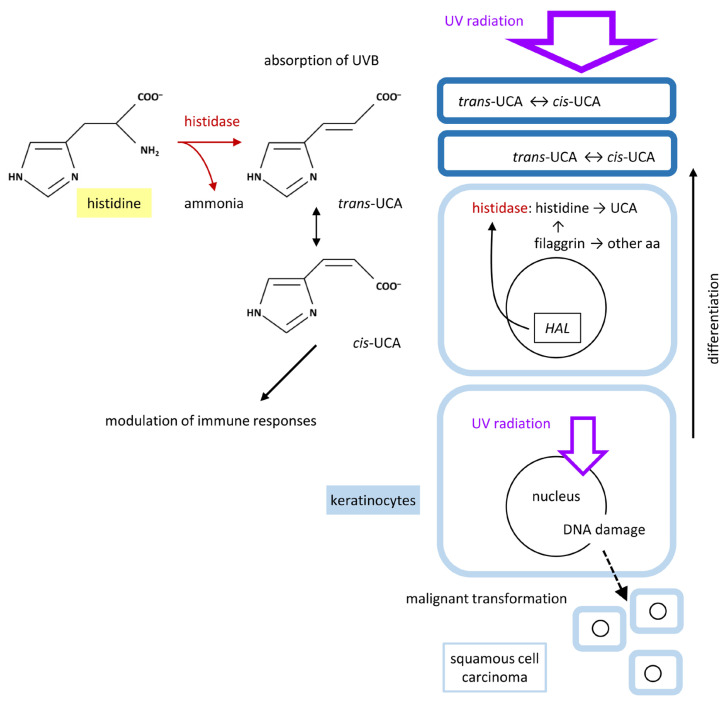
Histidase-mediated conversion of histidine into urocanic acid (UCA) and functions of UCA in epidermal keratinocytes. The expression of the *Histidine ammonia-lyase* (*HAL*) gene in differentiated epidermal keratinocytes provides the enzyme histidase, which catalyzes the conversion of histidine to *trans*-UCA, which mainly resides in the cornified layer of the epidermis. Thick blue lines indicate the cornified envelope of enucleate keratinocytes in this layer. Solar ultraviolet (UV) radiation is partly absorbed by UCA, leading to the reversible isomerization of *trans*- and *cis*-UCA. UCA-mediated absorption reduces the level of UV radiation reaching the lower layers of the epidermis and, thereby, also the risk of developing UV-induced skin cancer such as squamous cell carcinoma.

The catalytic activity of histidase generates *trans*-UCA in the granular layer and possibly also in the stratum corneum. In contrast to liver cells, which express both histidase and urocanase, an enzyme that catabolizes UCA to 4,5-dihydro-4-oxo-5-imidazolepropanoate, keratinocytes do not express urocanase. Accordingly, the formation of UCA in the keratinocytes leads to the accumulation of UCA in the uppermost layers of the epidermis. The rate of UCA formation is determined by the amount of histidase and the availability of the substrate, histidine, which depends on the expression level of filaggrin and the activity of proteases that degrade filaggrin (Figure 4). UCA is lost from the epidermis when corneocytes containing UCA are shed by the physiological process of desquamation and when the skin surface is exposed to water, leading to the extraction of water-soluble UCA [75]. UCA can also be degraded by commensal bacteria such as *Staphylococcus epidermidis* [76,77]. Notably, skin-penetrating parasitic nematodes utilize UCA as a chemoattractant [78].

UCA absorbs ultraviolet (UV) B radiation (280–320 nm) and to some extent also UVA radiation (320–380 nm). UV is more efficiently absorbed by UCA than by histidine or other free amino acids [75]. However, high amounts of amino acids within proteins contribute significantly to epidermal UV absorption [75]. UV irradiation causes the isomerization of UCA. The direct product of histidase is the trans isomer of UCA. UV converts *trans*-UCA to *cis*-UCA and vice versa. The presence of *cis*-UCA can be used as a marker of UV exposure of the skin [79].

It was suggested some time ago that UCA acts as an endogenous sunscreen [80]. Experimental evidence for the protective function of UCA was obtained in a study in normal and histidinemic mice, which contain only 10% of the normal concentration of UCA in the stratum corneum [28]. UVB irradiation of mice that were shaved on the back yielded significantly higher levels of epidermal DNA damage and cell death in histidinemic mice [28]. Similarly, the roles of proteolytic degradation of filaggrin as a source of histidine and UCA were supported by experimental studies [81,82].

In humans, the single nucleotide polymorphism rs721199 in the *HAL* gene (12q23.1) was identified as susceptibility locus for cutaneous squamous cell carcinoma (SCC), a UV-induced skin cancer [83]. Polymorphisms of *HAL* are also associated with the level of 25-hydroxy-vitamin D, which is formed in a UVB-dependent process in the skin [84,85]. In line with this association, carriers of *FLG* mutations have decreased concentrations of epidermal UCA [86] and increased concentrations of 25-hydroxy-vitamin D [87].

The UV-mediated isomerization of *trans*-UCA to *cis*-UCA has a potential role in the initiation of the immunosuppressive effects of UV irradiation [88,89]. *cis*-UCA but not *trans*-UCA suppresses immune reactions [90,91,92]. The mechanism of action of *cis*-UCA and its main target cells are still under investigation [77,93,94].

## 5. Tyrosinase Controls the Polymerization of Tyrosine to Melanin

### 5.1. Tyrosinase and Related Proteins in the Control of Melanogenesis

**Figure 5:**
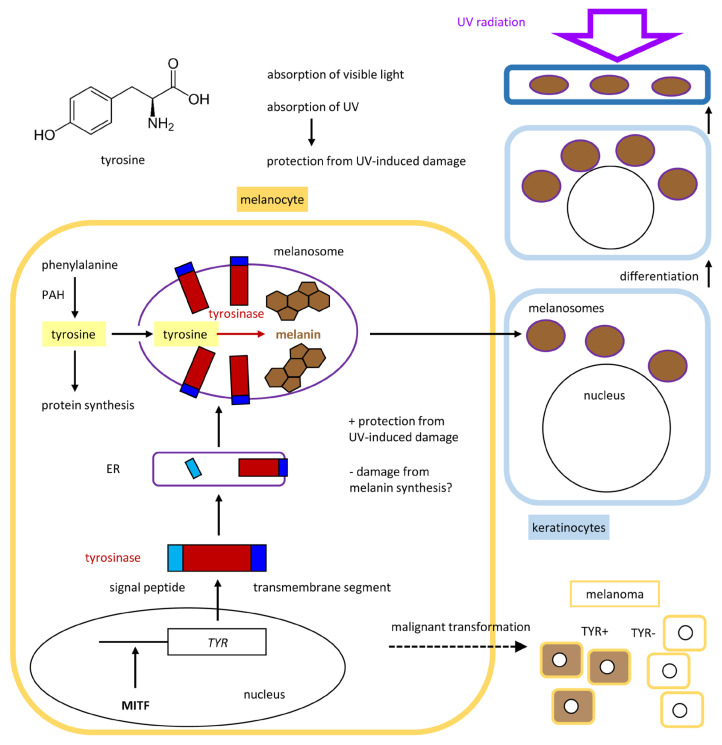
Tyrosine metabolism in melanocytes. The melanocyte-inducing transcription factor (MITF) activates the transcription of *tyrosinase* (*TYR*) in melanocytes. A classical signal peptide directs tyrosinase to the endoplasmic reticulum (ER), where it is anchored in the membrane. Enzymatic activity is restricted to lysosome-like organelles termed melanosomes, leading to the accumulation of melanin. Melanosomes are transferred to neighboring keratinocytes. Melanin absorbs radiation in the visible and ultraviolet ranges of wavelengths, contributing to visible pigmentation and protection against UV-induced damage. PAH, phenylalanine hydroxylase.

The role of tyrosine as a precursor of melanin is probably the most well-characterized non-proteinogenic function of an amino acid in the skin. The synthesis of melanin is confined to melanocytes because of the confined expression of the molecular drivers of melanogenesis, of which the enzyme tyrosinase is the most critical factor. Tyrosinase is encoded by the *TYR* gene and has tyrosine hydroxylase and dopa oxidase activities. It catalyzes the rate-limiting steps of melanogenesis (Figure 5), which involves the oxidation of tyrosine to dopaquinone and subsequent reactions leading to its polymerization. Tyrosinase-related protein 1 (TYRP1, 5,6-dihydroxyindole-2-carboxylic acid oxidase) and L-dopachrome tautomerase (DCT, also known as TYRP2) catalyze further reactions. TYR, TYRP1 and DCT/TYRP2 have the same protein organization with a signal peptide, a tyrosinase domain and a transmembrane segment, leading to co-localization as membrane-anchored proteins within specialized organelles, known as melanosomes. Tyrosinase and its molecular relatives bind two copper ions via two sets of three histidine residues.

The genes *TYR*, *TYRP1* and *DCT* are activated by the microphthalmia-associated transcription factor, also known as the melanocyte-inducing transcription factor (MITF) [95,96]. This transcription factor is expressed specifically in melanocytes and cells of the retinal pigment epithelium (RPE) [97]. Accordingly, melanin is synthesized in melanocytes and the RPE. Mutations of *TYR* cause oculocutaneous albinism type 1 (OCA1), which is subdivided in tyrosinase-negative OCA1A and OCA1B, which is characterized by mutations that allow residual tyrosinase activity.

Within melanocytes, melanogenesis takes place in membrane-enclosed melanosomes, ensuring a physical separation of these reactions from critical processes in other organelles and the cytoplasm [98]. Pre-melanosome protein (PMEL) forms a fibrillar matrix on which melanin polymerization takes place. Upon accumulation of melanin and structural maturation, melanosomes are transferred to the neighboring keratinocytes [99], where they absorb UV radiation to prevent damage to the DNA and other cell components. Melanosomes are maintained as keratinocytes differentiate and move toward the skin surface, thereby facilitating high levels of UV absorption in the layers above the stem cells and proliferating cells located in the basal layer of the epidermis. Thus, melanocytes residing in the basal layer are protected by their own melanosomes and by melanosomes in suprabasal keratinocytes.

### 5.2. Tyrosine Is the Main Precursor of Melanin

**Figure 6:**
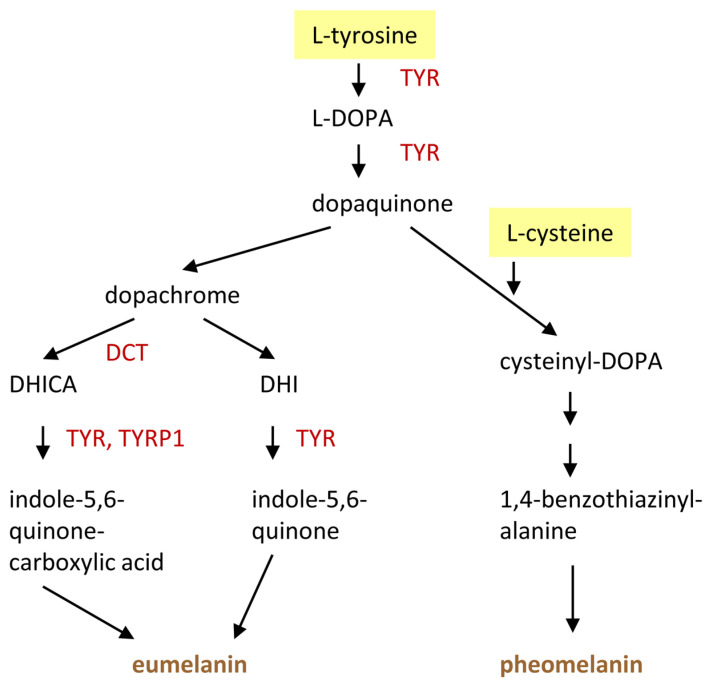
Tyrosine and cysteine are substrates of melanin synthesis. Key steps in the synthesis of eumelanin and pheomelanin are depicted. The enzymes are presented by their gene symbols. DCT, dopachrome tautomerase; L-DOPA, L-3,4-dihydroxyphenylalanine; TYR, tyrosinase; TYRP1, tyrosinase-related protein 1.

The molecular process of melanogenesis has been reviewed extensively [100,101,102]. Here, we highlight only aspects related to the amino acid metabolism. Tyrosine is a non-essential amino acid that is nevertheless mainly obtained from the diet. The mechanism of transport of tyrosine into melanocytes, possibly involving SLC7A5/LAT1, has not been fully characterized yet [102]. In addition, it is produced by hydroxylation of the essential amino acid phenylalanine. The responsible enzyme phenylalanine hydroxylase (PAH) is mainly expressed in the liver, but it has also been detected in melanocytes [103]. Tyrosinase catalyzes the hydroxylation of L-tyrosine to L-dihydroxyphenylalanine (L-DOPA) and the subsequent formation of L-dopaquinone. In the presence of low concentrations of cysteine, eumelanin—a dark brown to black pigment—is produced via several intermediates, involving non-enzymatic reactions as well as enzyme-catalyzed steps. Dopachrome tautomerase (DCT) catalyzes the conversion of dopachrome into 5,6-dihydroxyindole-2-carboxylic acid (DHICA), which subsequently polymerizes in a tyrosinase-catalyzed oxidative reaction. The latter reaction is also controlled by TYRP1 [102]. An alternative path leads from dopachrome via 5,6-dihydroxyindole and indole-5,6-quinone to polymerization and eumelanin formation. In the presence of a high concentration of cysteine, pheomelanin—a reddish-brown pigment—is produced via cysteinyl-DOPA and other intermediates [104] (Figure 6). The concentration and utilization of cysteine is determined by transporters, which are discussed in Section 7. Notably, tyrosinase is able to conjugate dopa with glutathione to form pheomelanin [105].

Genome-wide association studies (GWAS) have identified multiple polymorphisms in melanogenesis-related genes, indicating that melanin production protects against cutaneous malignant melanoma, squamous cell carcinoma and basal cell carcinoma [106]. However, there is an increasing amount of evidence for the differential roles of eumelanin and pheomelanin. While eumelanin reduces the risk of skin cancer through its UV-photoprotective properties, pheomelanin has negative effects. The synthesis of pheomelanin causes oxidative stress and UV-independent DNA damage [107,108]. Accordingly, both a lack of sufficient eumelanin and the direct effects of pheomelanogenesis appear to increase the risk of skin cancer in individuals having abundant pheomelanin [106].

## 6. Metabolic Reactions of Other Amino Acids in the Skin

### 6.1. Formation of Free Amino Acids by Hydrolysis of Proteins During Keratinocyte Cornification

Free amino acids are, to some extent, synthesized as part of the cellular metabolism [4,5], taken up from the extracellular milieu via specific transporter proteins (see Section 7) or generated by the catabolism of proteins [6]. Protein degradation by proteasomes or lysosomes, with or without the involvement of autophagy, is a major source of free amino acids in most cell types, including keratinocytes [109,110] and melanocytes [111]. Terminally differentiated keratinocytes have an additional major source of amino acids, the degradation of filaggrin and related proteins during and after cornification. Filaggrin is a large protein in which an amino-terminal S100 domain is followed by a repetitive region of low amino acid sequence complexity. Although previously known as a histidine-rich protein, filaggrin is actually particularly rich in serine and glycine, but other amino acids are also abundant because of the length of the protein beyond 3000 residues. The proform of filaggrin is proteolytically degraded in a stepwise manner, leading to the appearance of defined filaggrin units, peptides and, ultimately, free amino acids. The latter are important components of the NMF [112,113,114]. Mutations of *filaggrin* (*FLG*) are associated with atopic dermatitis [115].

Raman confocal micro-spectroscopy allows the estimation of free amino acid concentrations in the stratum corneum. Carriers of *FLG* mutations have a clearly altered amino acid profile in the cornified layer [86]. However, also in patients without filaggrin mutations, alanine, histidine and proline showed a significant negative correlation with the severity of atopic dermatitis [50].

### 6.2. Tryptophan Is Converted to a Ligand of the Aryl Hydrocarbon Receptor

Tryptophan is an aromatic amino acid that acts as a building block of proteins and a precursor of serotonin, melatonin and vitamin B3. Both tryptophan residues in proteins and free tryptophan efficiently absorb UVB radiation. UVB converts part of the tryptophan pool into 6-formylindolo [3,2-b]carbazole (FICZ), which is a ligand of the aryl hydrocarbon receptor (AHR) [116,117,118]. Furthermore, FICZ acts as photosensitizer with an absorbance maximum at 390 nm and may be useful for the photodynamic elimination of tumor cells in the skin [119,120]. AHR is also activated by other metabolites of tryptophan, such as kynurenic acid [121] and indole-3-aldehyde [25], which require further investigation in the skin [26,122].

### 6.3. Glutamine Is Converted to Pyrrolidone Carboxylic Acid, a Component of the NMF

Glutamine is an important amino acid in epidermal protein synthesis, because glutamine and lysine residues serve as sites of transglutaminase-mediated isopeptide formation. Transglutaminase-mediated protein cross-linking is central to epidermal cornification, and the glutamine-rich protein involucrin is a main structural building block of the cornified envelope [123]. Glutamine is the most abundant free amino acid in the blood and an important source of cellular energy. It is involved in the synthesis of purines and is therefore critical for proliferating cells. Besides glycine and cysteine, glutamine is also involved in the gamma-glutamyl cycle, which produces the important redox regulator glutathione (γ-L-glutamyl-L-cysteinylglycine). One of the molecules of the gamma-glutamyl cycle, pyrrolidone carboxylic acid (PCA), also known as pyroglutamic acid or 5-oxoproline, accumulates in the stratum corneum. PCA was proposed to be formed non-enzymatically in the epidermis [124]. However, it is noteworthy that the expression of gamma-glutamylcyclotransferase (GGCT), which catalyzes the formation of PCA from gamma-glutamyl dipeptides, is upregulated during terminal differentiation of keratinocytes (https://www.proteinatlas.org/ENSG00000006625-GGCT/tissue/skin#img, last accessed on 27 June 2025) [125]. The reaction catalyzed by GGCT is a step in the catabolism of glutathione. Thus, the expression of GGCT in the granular layer may alter the redox status of differentiated keratinocytes to allow oxidative reactions and concomitantly produce PCA as a component of the NMF in the stratum corneum. Notably, another enzyme participating in the degradation of glutathione, gamma-glutamyltransferase 6 (GGT6), is also elevated in differentiated keratinocytes [125]. The metabolism of glutamine appears to be a particularly promising topic of further research into the homeostasis of the epidermis.

### 6.4. Serine Regulates Epidermal Cell Proliferation

Serine is not an essential dietary amino acid. However, it was recently shown that epidermal keratinocytes depend on the uptake of extracellular serine [126]. Serine is converted by serine hydroxymethyltransferase (SHMT) into glycine and tetrahydrofolate-bound one-carbon units, which are essential for the proliferation of keratinocytes. The pharmacological inhibition of SHMT suppressed both epidermal cell proliferation and inflammation in a mouse model of psoriasis, suggesting that the serine/glycine metabolism is a promising target for the treatment of inflammatory hyperproliferative skin diseases [126]. A similar strategy is being tested for the therapy of cancers [127,128,129].

Recently, a critical role of serine in the response to skin injury was reported [22]. High levels of serine are required for the growth of hair, whereas low levels thereof cause hair follicle stem cells to support the repair of the skin epithelium [22]. Furthermore, serine is a major component of the NMF, and roles of serine in skin aging, the control of skin microbes and cutaneous immune defenses have been proposed [130,131,132,133].

## 7. Amino Acid Transporters in the Skin

Besides chemical reactions increasing or decreasing the concentrations of amino acids in specific compartments, the transport of amino acids across membranes is an important and tightly controlled part of the amino acid metabolism in the skin. Amino acid transporters are membrane-bound proteins that transfer amino acids into and out of organelles or cells [5]. Generally, the transport of amino acids is complex because the relationships between transporters and substrates are not monospecific. This means that a particular amino acid can be transported by more than one transporter [134]. The L-type amino acid transporter (LAT) 1 is the major transporter of histidine and large neutral amino acids, such as phenylalanine, leucine, isoleucine, tryptophan and tyrosine, with additional roles in the transport of carboxylic acids, thyroid hormone and xenobiotics. Other transporters have a narrower substrate range. For example, SLC7A11 is specific for cystine and glutamic acid. As the expression profile of transporters is only incompletely known, the precise amino acid transport pathways depending on keratinocytes in the skin are not fully understood at present. Many but not all amino acid transporters belong to the superfamily of solute carrier (SLC) proteins and especially to the families of SLC1, SLC6 and SLC7 proteins [135,136,137]. Here, we mention only a few amino acid transporters that have been discussed in recent papers related to the skin.

LAT1/SLC7A5 was implicated in the etiology of psoriasis. Deletion of *Slc7a5* in γδ and CD4 T cells reduced the psoriasis-like symptoms in imiquimod-treated mice [138,139]. Deletion of *Slc7a5* in keratinocytes did not have an effect in this model, suggesting that LAT2/SLC7A8 is more important than LAT1/SLC7A5 in epithelial skin cells [138,139]. LAT1/SLC7A5 and LAT2/SLC7A8 are upregulated in basal cell carcinoma [140]. LAT1/SLC7A5 was identified as a prognostic factor for poor outcome of cutaneous melanoma [141]. Interestingly, the expression levels of the amino acid transporters SLC1A4, SLC1A5, SLC3A2, SLC6A9, SLC7A1, SLC7A2, SLC7A5, SLC7A7, SLC7A11, SLC38A1, SLC38A2 and SLC43A1, and the concentrations of glycine, alanine and leucine, are decreased in epidermal keratinocytes undergoing UVB-induced senescence [142]. SLC16A10, also known as aromatic amino acid transporter 1 and T-type amino acid transporter 1 (TAT1), transports phenylalanine into melanosomes and promotes melanogenesis [143]. SLC15A4 facilitates the proton-coupled transmembrane transport of histidine and oligopeptides out of lysosome into the cytosol. Genome-wide association studies (GWAS) have identified SLC15A4 as a susceptibility locus of systemic lupus erythematosus [144]. A recent study showed that SLC15A4 is a polyamine transporter [136].

SLC7A11, also known as xCT, is a sodium-independent and chloride-dependent cystine-glutamate antiporter. A recent report showed that SLC7A11 is directly regulated by p63, a master regulator of ectodermal development and keratinocyte differentiation [145]. Accordingly, it is possible that dysregulation of SLC7A11 in carriers of p63 mutations contributes to the etiology of ankyloblepharon–ectodermal defects–cleft lip/palate (AEC) syndrome [145]. SLC7A11 was also suggested to act as an unconventional H^+^ transporter in lysosomes [146], a role that remains to be investigated in the skin.

SLC45A2 is a proton/glucose exporter that is located in melanosomes. Targeted *Slc45a2* gene ablation leads to enhanced glycolysis and acidification of melanosomes, thereby impairing melanogenesis [147]. In human patients, mutations of SLC45A2 cause oculocutaneous albinism type 4 [148]. Another member of this large family of transporter proteins, SLC24A5, also known as sodium/potassium/calcium exchanger 5 (NCKX5), is involved in skin pigmentation. Homozygous or compound heterozygous mutations in the *SLC24A5* gene cause oculocutaneous albinism type 6 (OCA6) [149].

The major facilitator superfamily domain-containing protein 12 (MFSD12) (CDD: 341044) transports cysteine into lysosomes and melanosomes. Thereby, MFSD12 controls the levels of the oxidized dimer of cysteine, known as cystine, and cysteinyl-dopas, which are required for the synthesis of pheomelanin in melanosomes [111]. Cystinosin (CTNS), a seven-transmembrane domain protein (domain CDD: 130026), transports cystine out of lysosomes. Furthermore, lysosomal proteolysis is likely to contribute to the production of free cysteine in lysosomes [111].

## 8. Conclusions

In this review, we have discussed special features of the cutaneous amino acid metabolism with a focus on the epidermis. Amino acids are utilized in the synthesis of proteins that form the largest portion of the protective layer on the surface of the body, the stratum corneum. Conversely, the breakdown of specific proteins in differentiated keratinocytes releases amino acids, which largely remain present in their free form within the stratum corneum where they contribute to the moisturization of the skin surface. Arginine and histidine are metabolized by enzymes, arginase 1 and histidase, that are specifically expressed in differentiated keratinocytes prior to cornification. Among their metabolites, urocanic acid is best characterized as an endogenous sunscreen and modulator of immune reactions. Another highly skin-enriched enzyme of the amino acid metabolism is tyrosinase, the key regulator of pigment formation in melanocytes. Tyrosine conversion into eumelanin is critical for protection against UV irradiation, whereas conversion of tyrosine and cysteine into pheomelanin can have harmful effects due to enhanced oxidative stress. Further enzyme-dependent and independent reactions of amino acids and the transport of amino acids through different skin compartments contribute to the control of the protective functions, the homeostasis and the regeneration of the epidermis.

## 9. Future Directions

Although some aspects of cutaneous amino acid metabolism, such as the fundamental importance of the conversion of tyrosine to melanin in pigment cells and the production of free amino acids by the catabolism of filaggrin, are well characterized, the chemical reactions, the transport and the functions of many amino acids and amino acid-derived metabolites require more investigation. For example, the roles of UCA in UVB-dependent immunomodulation are still not entirely understood. The peculiar expression pattern of arginase 1 suggests an as-yet-unknown function in normal skin. The roles of free amino acids and amino acid-derived metabolites in the NMF require further stringent experimental tests. Furthermore, the roles of the skin microbiome in the degradation and conversion of amino acids have remained underinvestigated. Future studies of the cutaneous amino acid metabolism have a great potential to contribute to improvements in skin care, cosmetics and the management of skin diseases.

## Data Availability

Not applicable.
